# Chiroptical activity of Au_13_ clusters: experimental and theoretical understanding of the origin of helical charge movements†

**DOI:** 10.1039/d0na00833h

**Published:** 2020-11-05

**Authors:** Yukatsu Shichibu, Yuri Ogawa, Mizuho Sugiuchi, Katsuaki Konishi

**Affiliations:** Graduate School of Environmental Science, Hokkaido University North 10 West 5 Sapporo 060-0810 Japan shichibu@ees.hokudai.ac.jp konishi@ees.hokudai.ac.jp; Faculty of Environmental Earth Science, Hokkaido University North 10 West 5 Sapporo 060-0810 Japan

## Abstract

Ligand-protected gold clusters with an asymmetric nature have emerged as a novel class of chiral compounds, but the origins of their chiroptical activities associated with helical charge movements in electronic transitions remain unexplored. Herein, we perform experimental and theoretical studies on the structures and chiroptical properties of Au_13_ clusters protected by mono- and di-phosphine ligands. Based on the experimental reevaluation of diphosphine-ligated Au_13_ clusters, we show that these surface ligands slightly twist the Au_13_ cores from a true icosahedron to generate intrinsic chirality in the gold frameworks. Theoretical investigation of a monophosphine-ligated cluster model reproduced the experimentally observed circular dichroism (CD) spectrum, indicating that such a torsional twist of the Au_13_ core, rather than the surrounding chiral environment by helically arranged diphosphine ligands, contributes to the appearance of the chiroptical response. We also show that the calculated CD signals are dependent on the degree of asymmetry (torsion angle between the two equatorial Au_5_ pentagons), and provide a visual understanding of the origin of helical charge movements with transition-moment and transition-density analyses. This work provides novel insights into the chiroptical activities of ligand-protected metal clusters with intrinsically chiral cores.

## Abstract 2

The profound chiroptical nature of Au_13_ clusters was revealed from systematic experimental and theoretical investigations.

## Graphical abstract

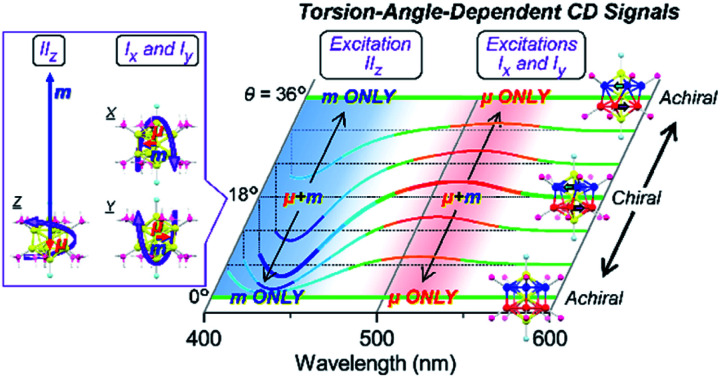

## Introduction

Among the various metal clusters reported to date, the geometric structures of gold clusters protected by thiolate^1^ and phosphine^2^ ligands have been well-defined through intensive X-ray crystallographic investigations, promoting experimental studies into chiral gold clusters.^3^ Structurally, the nature of a chiral gold cluster can be categorized as follows:^4^ (1) achiral gold cores inside chiral environments (category I), or (2) intrinsically chiral gold cores (category II). With respect to this categorization, various theoretical^5^ and numerical^6^ studies have aided the understanding of the origin of their unique chiroptical activities. However, the correlation between their structural details and chiroptical activities remains ambiguous due to the structural diversity and/or complexity of these clusters that mask the profound nature of the chiroptical responses (*i.e.*, a helical movement of charge density in an electronic transition, hereinafter referred to as a helical charge movement).^7^

**Scheme 1:**
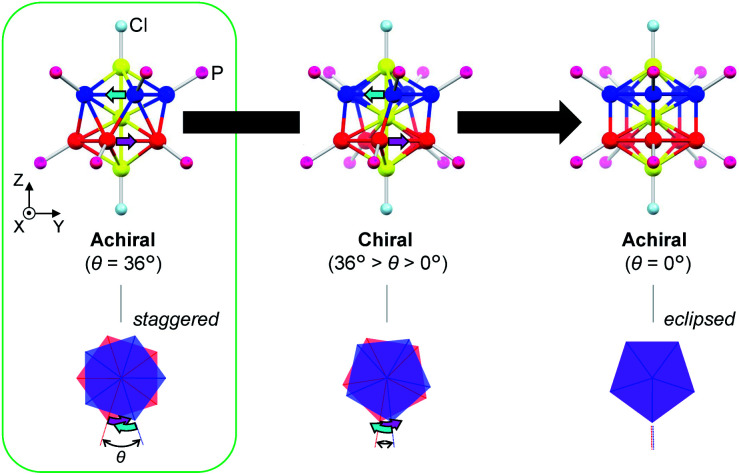
Geometric transformation of the Au_13_P_10_Cl_2_ framework upon variation in *θ* (the torsion angle between the two equatorial Au_5_ pentagons) from 36° (staggered conformation) to 0° (eclipsed conformation) *via* chiral *P*-helical conformations.

**Fig. 1:**
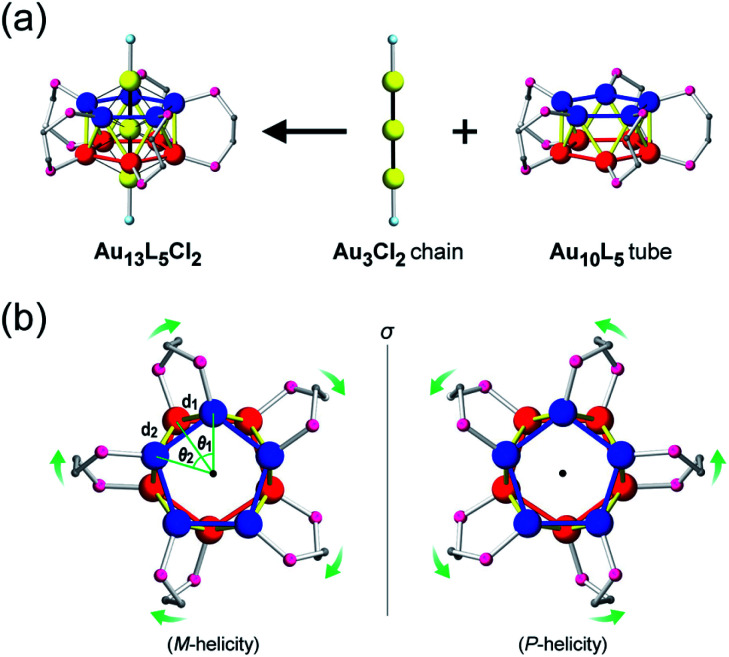
(a) Structural evolution to the [Au_13_L_5_Cl_2_]^3+^ cluster (*P*-helicity) and (b) top view of a pair of enantiomeric Au_10_L_5_ tubes. The dihedral angles (*θ*_1_ and *θ*_2_) are defined between two adjacent planes of Au_3_ triangles from two axial chloride-coordinated gold atoms and equatorial phosphorus-coordinated gold atoms. The Au–Au distances (*d*_1_ and *d*_2_) are defined between two adjacent gold atoms from the two five-membered Au_5_ rings. Color codes: yellow/blue/red spheres, Au; pink spheres, P; aqua spheres, Cl; gray spheres, C. For clarity, the other C and H atoms are omitted.

In this paper, we focus on icosahedral Au_13_ clusters having ten phosphorus and two chloride atoms (*i.e.*, the [Au_13_P_10_Cl_2_]^3+^-type clusters), and demonstrate the chiroptical responses depending on the transformation of the icosahedral Au_13_ core with variation in *θ*, the torsion angle between the two equatorial Au_5_ pentagons (Scheme 1). Since the first structural report on a PMe_2_Ph-ligated Au_13_ cluster, namely [Au_13_(PMe_2_Ph)_10_Cl_2_]^3+^, by Mingos *et al.*,^8^ the icosahedral Au_13_ framework, which is ubiquitously found in ligand-protected gold clusters,^9^ has attracted increasing interest as a symbolic core of metal clusters, while the monophosphine-ligated Au_13_ cluster itself is unstable. By using a C2-bridged diphosphine ligand (L) of achiral DPPE (1,2-bis(diphenylphosphino)ethane), we previously succeeded in the synthesis and structural determination of a [Au_13_L_5_Cl_2_]^3+^ cluster (1; L = DPPE),^10^ providing facile access to chemically stable Au_13_ clusters protected by various diphosphine ligands.^11^ Recently, the [Au_13_L_5_Cl_2_]^3+^ cluster protected by chiral DIPAMP (1,2-bis[(2-methoxyphenyl)phenylphosphino]ethane), a derivative of DPPE (2-*R* or 2-*S*; L = *R*,*R*- or *S*,*S*-DIPAMP), was also synthesized and structurally determined.^12^ The common framework of 1 and 2 can be viewed as a rotaxane-like motif, *i.e.*, threading of a linear Au_3_Cl_2_ chain through a pentagonal antiprismatic Au_10_L_5_ tube (Fig. 1a). This tube contains two staggered Au_5_ rings, which are cross-linked by five diphosphine ligands exhibiting a helical arrangement. As shown in Fig. 1b, the tube can be presented in the *P*- or *M*-helicity, depending on the manner of the helical arrangements (*i.e.*, right- and left-handed, respectively).

In terms of the Au_13_ cores, the Au_13_ core bearing an icosahedral (*θ* = 36°) or a bicapped pentagonal prismatic (*θ* = 0°) structure is achiral, while that with other values of *θ* (*i.e.*, 36° > *θ* > 0°) is chiral (Scheme 1). With respect to the chirality of the [Au_13_L_5_Cl_2_]^3+^ cluster, Li *et al.* claimed that 1 should be chiral because of the helical arrangement of its achiral ligands (belonging to category I), although the enantiopure product of 1 was not obtained by chiral HPLC due to the rapid racemization.^13^ Thereafter, Zhan *et al.* reported that the enantiopair of 2 possessed chiral twisted Au_13_ cores induced by the helically arranged chiral ligands (belonging to category II).^12^ However, it was uncertain whether the helically arranged achiral ligands in 1 also induced such a torsional twist in the Au_13_ core. Moreover, if such achiral (1) and chiral (2) ligands both induce the intrinsic chirality to the Au_13_ cores, any correlation between the torsion of the Au_13_ framework and circular dichroism (CD) signals remains elusive, which limits a comprehensive understanding of their chiroptical origins, including the above-mentioned helical charge movements. Thus, to examine the effect of surface diphosphine ligands on the torsional twist of the Au_13_ cores, we herein report an experimental reevaluation of the [Au_13_L_5_Cl_2_]^3+^ clusters (1 and 2). Furthermore, we also report a theoretical investigation into a simplified model, *i.e.*, the [Au_13_(PH_3_)_10_Cl_2_]^3+^ cluster, where the torsion angle *θ* was varied (Scheme 1) to evaluate the effect of the torsion angle on the CD signals. Based on a systematic approach using transition-moment and transition-density analyses, we theoretically demonstrate a torsion-angle-dependent chiroptical response and provide a visual understanding of the origin of helical charge movements in the Au_13_ cluster.

## Results and discussion

### Experimental structural reevaluation

**Fig. 2:**
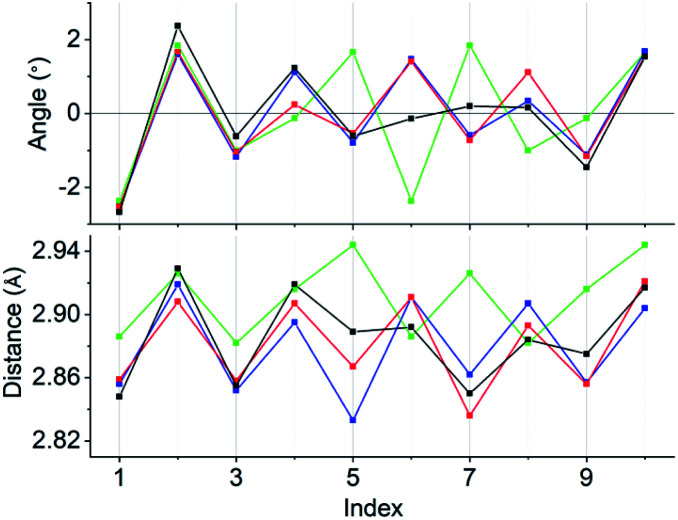
Line graphs of the angles (*θ*_1_ − 36 and *θ*_2_ − 36) and distances (*d*_1_ and *d*_2_) *vs.* the index numbers of 1 (black), 2-*R* (red), 2-*S* (blue), and [Au_13_(PMe_2_Ph)_10_Cl_2_]^3+^ (light green). Numbering of the indices is in accordance with Fig. S3.† Except for [Au_13_(PMe_2_Ph)_10_Cl_2_]^3+^, the “angles and distances” for the odd and even index numbers correspond to “*θ*_1_ − 36 and *d*_1_” and “*θ*_2_ − 36 and *d*_2_”, respectively.

Concerning the [Au_13_L_5_Cl_2_]^3+^ clusters (Fig. 1), torsion between the two staggered Au_5_ rings was reported only for the Cl salts of 2.^12^ Hence, we independently investigated the geometries of the PF_6_ salts of 1 and 2. Based on single-crystal X-ray structural analyses, it was apparent that 1 was obtained as a racemic compound,^10^ while 2-*R* and 2-*S* were obtained in their *P*- and *M*-helicities, respectively (Fig. S1 and S2†). The effect of the diphosphine ligands on the torsion of the Au_13_ cores was then revealed from a statistical analysis of the dihedral angles (*θ*_1_ and *θ*_2_) and the Au–Au distances (*d*_1_ and *d*_2_) of the Au_10_L_5_ tubes (Fig. 1b). For example, as was reported for 2,^12^ the dihedral angles relating to the cross-linked diphosphines (*θ*_1_) were narrower than the adjacent angles (*θ*_2_) (Fig. S3b and c†). The average angles of *θ*_1_ − 36 for 2-*R* and 2-*S* were −1.19 and −1.24°, respectively, which are ∼2° smaller than *θ*_2_ − 36 (Table 1). In addition, Au–Au distances related to the cross-linked diphosphines (*d*_1_) were shorter than the adjacent distances (*d*_2_) (Fig. S3b and c†), and the average distances of *d*_1_ for 2-*R* and 2-*S* were shorter by 0.053 and 0.055 Å than those of *d*_2_ (Table 1). These perfect periodic changes in the angles and distances for both enantiomers of 2 are reflected in the zigzag patterns that appear in Fig. 2 (red and blue lines). In the case of 1, such zigzag patterns were partially ambiguous (Fig. 2, black lines). In this regard, compared with 2, the differences in the average angles (*θ*_2_ − *θ*_1_) and distances (*d*_2_ − *d*_1_) became smaller, and the standard deviations of the angles and distances became larger (Table 1). In contrast, in the PMe_2_Ph-ligated Au_13_ cluster,^8^ which does not feature cross-linking between the two Au_5_ rings, such zigzag periodicities were disrupted (Fig. 2, light green lines), and their standard deviations were approximately doubled (Table 1). Therefore, these data clearly indicate that the helically arranged cross-linkage imparted by the C2-bridged diphosphine ligands induces a torsional twist between the two Au_5_ rings of the Au_10_L_5_ tubes by ∼1° from the ideal staggered angle (36°). In addition, the degree of torsion is higher in DIPAMP than in DPPE, which indicates the higher structural rigidity of 2.

**Table 1:** Average angles (*θ*_1_ − 36 and *θ*_2_ − 36) and distances (*d*_1_ and *d*_2_) of the Au_13_ clusters (with standard deviations)^a^

	*θ*_1_ − 36 (°)	*θ*_2_ − 36 (°)	*d*_1_ (Å)	*d*_2_ (Å)
1	−1.03 ± 0.97 [−2.67, +0.20]	+1.03 ± 0.92 [−0.14, +2.37]	2.863 ± 0.016 [2.848, 2.889]	2.908 ± 0.017 [2.884, 2.929]
2-*R*	−1.19 ± 0.70 [−2.52, −0.54]	+1.19 ± 0.51 [+0.24, +1.66]	2.855 ± 0.010 [2.836, 2.867]	2.908 ± 0.009 [2.893, 2.921]
2-*S*	−1.24 ± 0.68 [−2.54, −0.59]	+1.24 ± 0.49 [+0.34, +1.68]	2.852 ± 0.010 [2.833, 2.862]	2.907 ± 0.008 [2.895, 2.919]
[Au_13_(PMe_2_Ph)_10_Cl_2_]^3+^^b^	0.00 ± 1.60 [−2.37, +1.84]		2.911 ± 0.024 [2.882, 2.944]	

a The minimum and maximum values are given in square brackets.

b Data for *θ* − 36 and *d* in Fig. S3d are presented, since there is no concept of such differences in angles (*θ*_1_ and *θ*_2_) and distances (*d*_1_ and *d*_2_) because of the lack of cross-linked diphosphine ligands.

### Experimental optical spectra

**Fig. 3:**
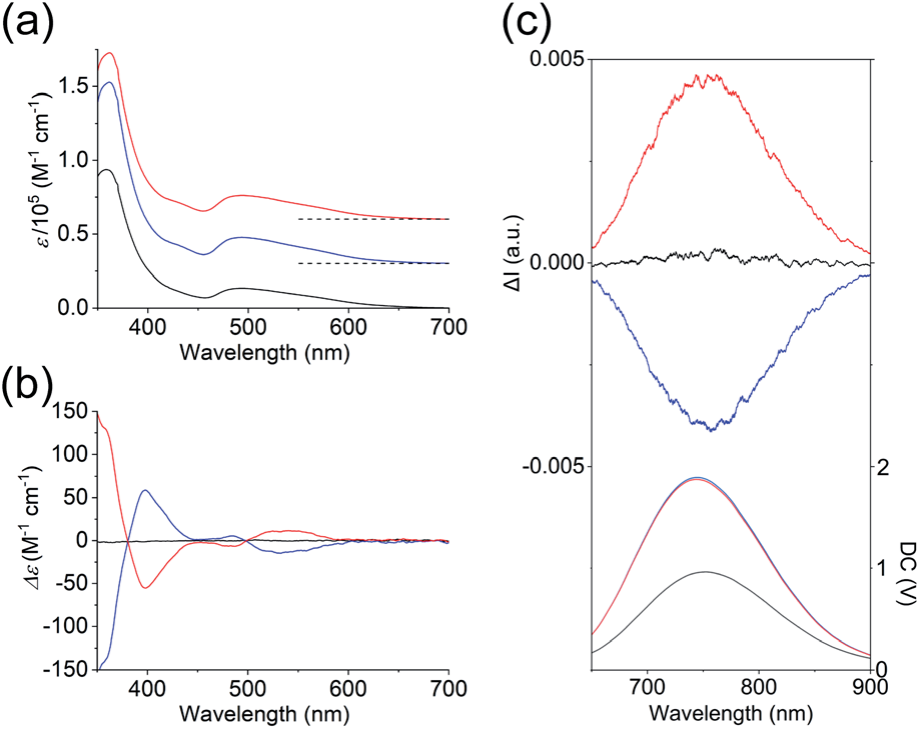
(a) Absorption, (b) CD, and (c) CPL (top) and PL (bottom) spectra (*λ*_ex_ = 495 nm) of the PF_6_ salts of 1 (black), 2-*R* (red) and 2-*S* (blue) in acetonitrile. The CPL and PL spectra measured by using a CPL apparatus are uncorrected. Corrected PL spectra are given in Fig. S4.† Baselines of the absorption spectra are shown by dashed lines in (a).

As shown in Fig. 3a, the absorption spectra of the PF_6_ salts of 1 and 2 were quite similar, exhibiting broad bands at ∼495 nm and intense bands at 360 nm. Previous theoretical studies into the absorption spectra of their Au_13_ clusters^12,14^ indicated that the broad bands at 495 nm were mainly attributed to Au(6sp) → Au(6sp) intraband transitions, while metal–metal transitions (*e.g.* Au(5d) → Au(6sp)) contributed to the band at 360 nm. This means that the contribution of the metal-based transitions within the Au_13_ cores to the near-UV-to-visible absorptions is large. In addition, the CD spectra of the 2-*R* and 2-*S* enantiomers showed perfect mirror images (Fig. 3b), whereby 2-*R* exhibited a positive first Cotton effect at 535 nm and negative Cotton effects at 485 and 398 nm. Previously, the experimentally obtained CD spectrum of the Cl salt of 2-*R* was theoretically well reproduced from the full structure of 2-*R*,^12^ and the calculated CD spectrum of the ligand-simplified structure of 1 was relatively similar to the experimental spectrum of 2-*R*.^13^ As these CD responses were connected to the metal-based transitions at approximately 400–600 nm, it is quite reasonable to assume that torsion between the two Au_5_ rings of the Au_13_ cores contributes to the chiroptical activities of the Au_13_ clusters, although we cannot ignore the effect of the surrounding chiral environments by the helically arranged achiral/chiral ligands on the CD responses.^4^

Several Au_13_ clusters, including 1, were reported to show relatively strong near-IR emissions,^10,11,15^ and we found that 2 also exhibited such an emission (Fig. S4†). In contrast to the emission peak of 1 at 818 nm, that of 2 at 792 nm was blue-shifted by 26 nm. In addition, the quantum yield of both enantiomers of 2 was 15%, which is 4% larger than that of 1.^14^ This can be attributed to the higher structural rigidity of 2 compared to 1, which can suppress nonradiative decay.^16^ Mirror images were also found in the circularly polarized luminescence (CPL) spectra of 2-*R* and 2-*S* (Fig. 3c), and the emission anisotropy factors at 760 nm (2.5 × 10^−3^ for 2-*R* and −2.3 × 10^−3^ for 2-*S*) were approximately one third of that of a chiral gold complex.^17^ It should also be noted here that the positive first Cotton effect at 535 nm (Fig. 3b, red line) and the positive CPL signal (Fig. 3c (top), red line) were both generated from 2-*R*, a Au_13_ cluster with *P*-helicity. The plus signs of these signals can be explained based on the exciton coupling theory^18^ and the excimer chirality rule^19^ for organic molecules with *P*-helicity. As these chiroptical spectroscopic techniques are sensitive to absolute structures, satisfaction of the above theory and rule may indicate a chiroptical analogy between organic molecules and gold clusters.

### Theoretical optical spectra

**Fig. 4:**
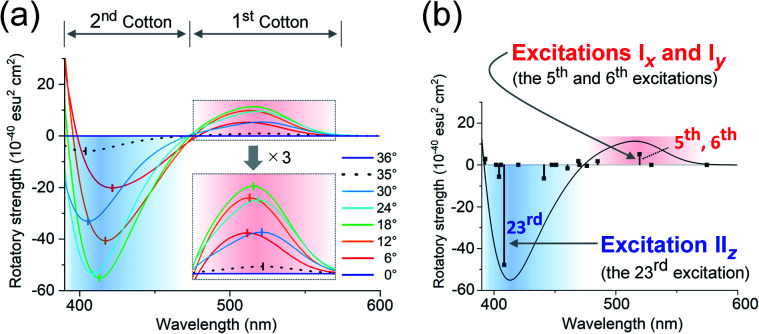
Calculated CD spectra of [Au_13_(PH_3_)_10_Cl_2_]^3+^ (a) upon variation in *θ* from 36° to 0°, and (b) at *θ* = 18°. Zones of the first and second Cotton effects are shaded in red and blue.

The experimental and theoretical observations of the chiroptical signals from the Au_13_ clusters, which contain icosahedral Au_13_ cores twisted slightly by ∼1°, motivated us to investigate the correlation between the CD signals and the torsion angles of the two Au_5_ rings of the Au_13_ core. To focus on the effect of the torsion of the Au_13_ core, we performed theoretical calculations of a ligand-simplified cluster model, *i.e.*, [Au_13_(PH_3_)_10_Cl_2_]^3+^, using various torsion angles (36° ≥ *θ* ≥ 0°; Scheme 1). The relative energy of the model at *θ* = 0° was more disfavored than that of the fully relaxed one (*θ* = 36°) by 1.32 eV, and the energy changes of the frontier orbitals at 36° ≥ *θ* ≥ 0° were small (Table S3†). The calculated absorption spectrum of the model at *θ* = 36° showed two bands at 520 and 462 nm, and the band at 520 nm was weakened upon decreasing the angle (Fig. S5†). In the calculated CD spectra, the Au_13_ clusters exhibiting staggered (*θ* = 36°) and eclipsed (*θ* = 0°) conformations of the two Au_5_ rings are silent, while the Au_13_ clusters with other values of *θ* present positive bisignate signals in the near-UV-to-visible region, where metal-based transitions mainly occur (Fig. 4a). This can be explained based on the symmetry of the Au_13_P_10_Cl_2_ framework (Scheme 1). More specifically, at 36° > *θ* > 0°, the Au_13_ cluster has *D*_5_ symmetry, which is chiral because of the lack of reflection (*S*_1_) and inversion (*S*_2_) symmetries.^7^ In contrast, the Au_13_ cluster bearing an icosahedral (*θ* = 36°) or a bicapped pentagonal prismatic (*θ* = 0°) structure exhibits higher *D*_5d_ or *D*_5h_ symmetries, respectively, which is achiral because of the presence of the *S*_*n*_ axis along the axial *z*-axis (*n* = 2 for *θ* = 36°, *n* = 1 for *θ* = 0°). As the value of *θ* decreases, a blue shift of the positive Cotton effect at ∼520 nm and a red shift of the negative Cotton effect at ∼410 nm were observed (Fig. 4a). In addition, the intensities of the positive and negative peaks, which exhibit gradual *θ*-dependent changes, reached their maximum and minimum values at *θ* = 18°, respectively. Interestingly, the theoretical CD spectrum of the Au_13_ model at *θ* = 35° (Fig. 4a, black dotted line) was similar to the experimental one of 2-*R* (Fig. 3b, red line) in terms of the peak shape and wavelength. In contrast, the intensity of the first Cotton effect at 522 nm of the model (*θ* = 35°) was +0.93 × 10^−40^ esu^2^ cm^2^ (Fig. 4a, black dotted line), which was approximately 1/350th of the theoretical intensity of that of 2-*R*.^12^ Such an enhancement effect on CD responses was theoretically demonstrated for several diphosphine-ligated gold clusters, where the chiral arrangement and/or nature of the ligands induced strong dissymmetric fields.^5*l*,*m*^ Therefore, these results clearly show that the torsion between the two Au_5_ rings of the icosahedral Au_13_ cores affects the CD responses of the Au_13_ clusters, and the helically arranged diphosphine ligands around the Au_13_ cores contribute to the significant enhancement of the responses.

### Transition-moment analysis

**Fig. 5:**
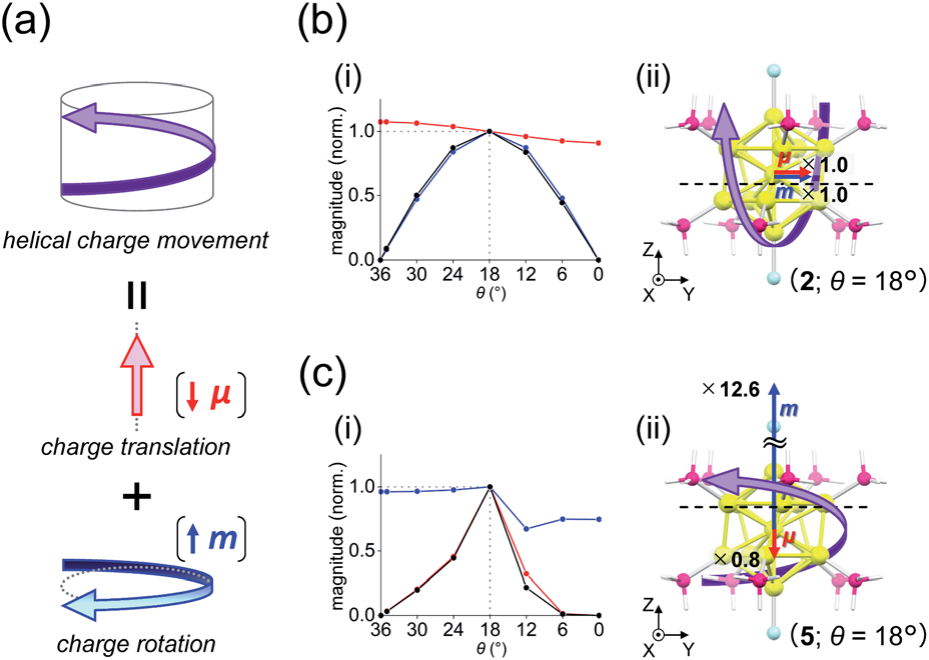
(a) Components of a helical charge movement. (i) Normalized magnitudes of the rotatory strength (black), electric transition dipole moment (red), and magnetic transition dipole moment (blue) at 36° ≥ *θ* ≥ 0°, and (ii) the electric (red) and magnetic (blue) transition dipole moments at *θ* = 18° for (b) excitation I_*y*_ and (c) excitation II_*z*_ of [Au_13_(PH_3_)_10_Cl_2_]^3+^. In parts b(ii) and c(ii), schematic representations of the helical charge movements are shown as helical purple arrows, the magnitudes of the moments relative to those for excitation I_*y*_ are given near the respective arrow tips, and dashed lines indicate the positions of the *xy*-cutplanes given in Fig. 6.

A key factor in determining chiroptical properties is the transition dipole moments. As shown in Fig. 5a, a helical charge movement in an electron transition requires a simultaneous charge translation and charge rotation, each of which induces electric and magnetic transition dipole moments (*i.e.*, ***μ*** and ***m***).^7^ Here, the term “charge” refers to the electron charge. From these two moments, the rotatory strength (*R*), which governs the CD intensity, is formulated using the following equation:

1 *R* = |***μ***||***m***|cos *α*

where *α* represents the angle between the moments. Thus, to understand the *θ*-dependent Cotton effects of [Au_13_(PH_3_)_10_Cl_2_]^3+^ (Fig. 4a), we analyzed the *θ*-dependences of *R*, ***μ***, and ***m***. In this study, we focused on the 5^th^, 6^th^, and 23^rd^ singlet excitations, because they dominate the positive first (515 nm) and negative second (413 nm) Cotton effects at *θ* = 18° (Fig. 4b and Table S5†), where the CD responses reached their maximum values (Fig. 4a).

For the 6^th^ excitation (hereafter excitation I_*y*_), |*R*| and |***m***| exhibited distinct parabolic relationships with *θ*, while |***μ***| was less dependent on *θ* (Fig. 5b(i)). As ***μ*** and ***m*** were oriented along the *y*-axis in parallel (*i.e.*, cos *α* ≈ 1) at 36° > *θ* > 0° (Fig. 5b(ii)), the *θ*-dependence of |*R*| was strongly coupled with the behavior of |***m***|, as indicated in eqn (1). Essentially the same features were observed along the *x*-axis (Fig. S8†) for the 5^th^ excitation (hereafter excitation I_*x*_), *i.e.*, the other primary excitation responsible for the positive Cotton effect at *θ* = 18° (Fig. 4b). For the 23^rd^ excitation, the primary excitation for the negative Cotton effect at *θ* = 18° (Fig. 4b), two types of excitations with *z*-axis-oriented transition moments (excitation II_*z*_ at *θ* ≥ 18° and excitation 
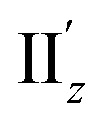
 at *θ* < 18°) were found from shape similarities of their transition densities (details are given in the subsequent section) and systematic shifts of their transition wavelengths. As shown in Fig. S9† (blue and orange lines), the transition wavelengths of excitations II_*z*_ and 
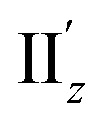
 showed blue and red shifts with decreasing *θ*, respectively, and overlap of the two excitations was observed at *θ* = ∼18°. A red shift of the negative Cotton effect with decreasing *θ* (Fig. 4a) can be attributed to such a complicated contribution of the two excitations. In contrast, a blue shift of the positive Cotton effect with decreasing values of *θ* (Fig. 4a) corresponds to the decrease in the transition wavelengths of excitations I_*x*_ and I_*y*_ with decreasing *θ* (Tables S6 and S7†). Excitation II_*z*_, including the 23^rd^ excitation at *θ* ≥ 18°, exhibited volcano-type relationships for |*R*| and |***μ***| with variation in *θ* (Fig. 5c(i)). Here, the *θ*-dependence of |*R*| was closely coupled to the behavior of |***μ***|, from eqn (1), because ***μ*** and ***m*** for excitation II_*z*_ were parallelly oriented at 18° > *θ* > 0° (*i.e.*, cos *α* ≈ 1) and antiparallelly oriented at 36° > *θ* ≥ 18° (*i.e.*, cos *α* ≈ −1) along the *z*-axis (Fig. 5c(ii)).

Table 2 shows excitation data at three representative values of *θ* for excitations I_*y*_ and II_*z*_ (entries 1–6). Entries 1–3 and 4–6 exhibited larger values of |***μ***| and |***m***| than their respective counterparts (*i.e.*, entries 4–6 and 1–3). At *θ* = 18°, the magnitudes of ***μ*** and ***m*** for excitation II_*z*_ (*i.e.*, entry 5) are 0.8 times smaller and 12.6 times larger than those for excitation I_*y*_ (*i.e.*, entry 2), respectively (Fig. 5b(ii) and c(ii), red and blue arrows). This significant difference in ***m*** leads to a large negative value of *R* in entry 5 (−47.87 × 10^−40^ esu^2^ cm^2^), the absolute value of which was more than nine times larger than that in entry 2 (5.095 × 10^−40^ esu^2^ cm^2^). Therefore, these data clearly indicate that the nature of the helical charge movements for excitations I_*y*_ and II_*z*_ at 36° > *θ* > 0°, representatively shown at *θ* = 18° as helical purple arrows in Fig. 5b(ii) and c(ii), is completely different.

**Table 2:** Selected singlet excitation data for [Au_13_(PH_3_)_10_Cl_2_]^3+^

Entry	*θ* (°)	*λ* (nm)	*R* (10^−40^ esu^2^ cm^2^)	|***μ***| (D)	|***m***| (*μ*_B_)
**Excitation I**_***y***_					
1	36	524	0.000	1.552	0.000
2	18	519	5.095	1.444	0.038
3	0	515	0.000	1.313	0.000

**Excitation II**_***z***_					
4	36	413	0.000	0.000	0.459
5	18	408	−47.87	1.082	0.477
6	0	402	0.000	0.000	0.356

### Transition-density analysis

**Fig. 6:**
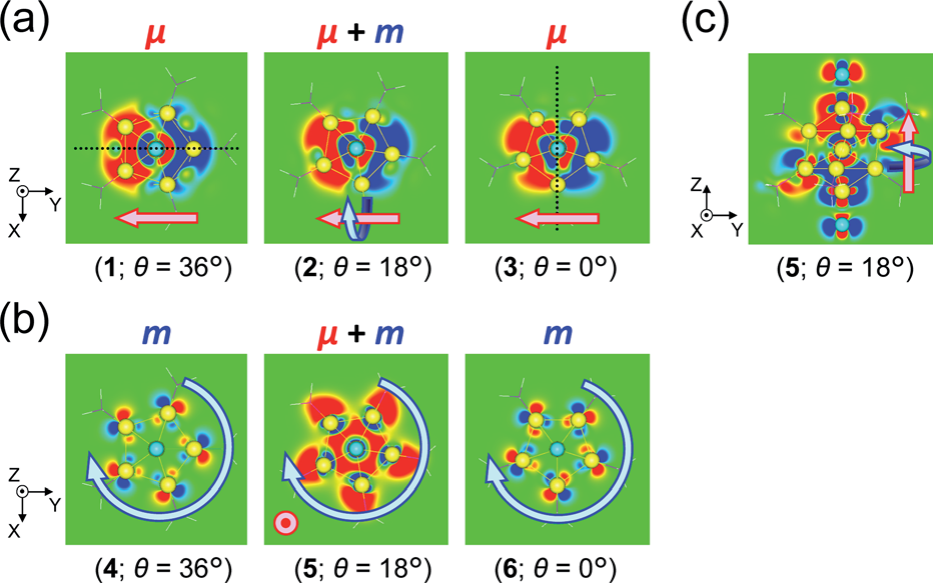
*xy*-Cutplane views of the transition densities for (a) excitation I_*y*_ and (b) excitation II_*z*_ of [Au_13_(PH_3_)_10_Cl_2_]^3+^ at three representative *θ* values (within ±0.00015 a.u. range). The red-colored and blue-colored areas represent electron increase and decrease during the electron excitations, respectively. Dotted black lines in part (a) represent the positions of a symmetric *yz*-plane (*θ* = 36°) and an antisymmetric *xz*-plane (*θ* = 0°). The positions of the *xy*-cutplanes in parts (a) (*z* = −0.25) and (b) (*z* = +1.0) are provided as dashed lines in Fig. 5b(ii) and c(ii), respectively. (c) *yz*-Cutplane view (*x* = 0.0) of entry 5. Pink and aqua arrows schematically indicate charge translations and rotations, respectively.

To obtain further insights into the origin of the helical charge movements for excitations I_*y*_ and II_*z*_, we investigated the generation factors of their charge translations and charge rotations (or ***μ*** and ***m***) using transition-density analysis. Fig. 6a shows *xy*-cutplane views of the three transition densities for excitation I_*y*_. At all angles (entries 1–3), wide red-colored (left side) and blue-colored (right side) areas were observed, which were also found in their overall views (Fig. S10a†). Thus, the charge translations from the blue to red areas, which are indicated by the (−*y*)-directed pink arrows in Fig. 6a, are the primary contributors to the (+*y*)-directed ***μ*** (Fig. 5b(ii)), and the reduced *θ*-dependence of |***μ***| for excitation I_*y*_ (Fig. 5b(i), red line) can be explained from the simultaneous observation of such similarly shaped areas in entries 1–3. The strong *θ*-dependence of |***m***| (Fig. 5b(i), blue line), on the other hand, appears to be related to the degree of asymmetry, which arises from the conformation of the two Au_5_ pentagons (Scheme 1). Specifically, the transition densities at *θ* = 36 and 0° have a symmetric *yz*-plane and an antisymmetric *xz*-plane containing the centred Au atoms, respectively (Fig. 6a, dotted lines). For these cases, the charge translation occurs only along the *y*-axis; hence, |***m***| reaches zero. However, at 36° > *θ* > 0°, the Au_13_ cluster model loses such symmetric planes, and the degree of asymmetry is expected to increase to its maximum at *θ* = 18°. These results indicate that the helical charge movement for excitation I_*y*_ at 36° > *θ* > 0° along the *y*-axis (*e.g.*Fig. 5b(ii)) was triggered by deformation of the “highly symmetric” *y*-axis-oriented charge translation (*θ* = 36° or 0°), which induces the charge rotation around the *y*-axis. Again, the same is essentially true along the *x*-axis for excitation I_*x*_ (Fig. S11†).

For excitation II_*z*_, the *xy*-cutplane views of the transition densities at *θ* = 36° and 0° (entries 4 and 6) are quite similar to one another, whereas that at *θ* = 18° (entry 5) is clearly different (Fig. 6b). In contrast, entries 4–6 all showed significantly larger |***m***| values (0.356–0.477 *μ*_B_), *i.e.*, by one order of magnitude, compared to entry 2 (0.038 *μ*_B_; Table 2). In the cases of entries 4 and 6, the charge rotations around the *z*-axis (Fig. 6b, aqua arrows), which arises from the circular patterns of the four-clover-leaf-like lobes on the Au_5_ rings (Fig. 6b and S10b,† left and right), are the primary contributors to the strong and (+*z*)-directed ***m*** (Fig. 5c(ii)). In entry 5, it is reasonable to assume that the charge rotation is essentially identical to those in entries 4 and 6, and the clear difference in transition density (Fig. 6b, middle) results from a mixture of excitations II_*z*_ and 
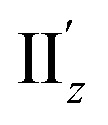
 because of their above-mentioned overlap at *θ* = ∼18° (Fig. S9†). This assumption is supported by the observation that the transition densities of entry 5 for excitation II_*z*_ (Fig. 6b and S10b,† middle; Fig. 6c) and entries 4′–6′ for excitation 
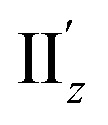
 (Fig. S12†) are similar to one another, all exhibiting wide red- and blue-colored areas in the ±*z* regions leading to the generation of ***μ*** along the *z*-axis (Fig. 6c and S12b†). Therefore, the helical charge movement for excitation II_*z*_ at 36° > *θ* > 0° along the *z*-axis (*e.g.*Fig. 5c(ii)) is generated by the combination of the strong charge rotation around the *z*-axis originating in the Au_5_ rings and the *z*-axis-oriented charge translation, the latter of which mainly comes from the other excitation (*i.e.*, excitation 
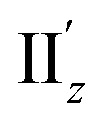
).

Finally, it should be noted that these theoretical analyses reveal the unique origin of ***m*** in the Au_13_ cluster, whereby the circularly arranged gold atoms around the cluster axis can generate a remarkably strong charge rotation around the axis in electronic transitions, which is significantly more effective than the charge rotation induced by the deformation of the highly symmetric gold core. With respect to understanding the structural origins of the chiroptical activities of gold clusters, previous theoretical and numerical studies have generally focused on the “asymmetric arrangement” of the gold atoms and/or the surrounding chiral environments.^5,6^ In contrast, our systematic theoretical approach based on the simple Au_13_ cluster model highlights the importance of the “circular arrangement” of gold atoms, and so could lead to a boost in chiroptical studies into metal clusters.

## Conclusions

We compared the geometric structures and chiroptical properties of achiral/chiral diphosphine-ligated Au_13_ clusters and revealed that cross-linking through diphosphine ligands induced small torsional twists between the two equatorial Au_5_ rings of the Au_13_ cores, contributing to their chiroptical properties. Furthermore, we systematically investigated the effect of torsion between the two equatorial Au_5_ rings of the Au_13_ cores on their chiroptical activities using theoretical calculations for a simplified Au_13_ cluster model. The enhancement of the chiroptical signals in the calculated circular dichroism spectra was observed in response to the degree of asymmetry of the Au_13_ model. In addition, transition-moment and transition-density analyses revealed the profound origin of the helical charge movements, and the unique origin of the strong charge rotation arising from the equatorial Au_5_ rings was demonstrated. Our results indicate that not only the conventional asymmetric arrangement but also the circular arrangement of the metal atoms can influence the chiroptical activities of ligand-protected metal clusters bearing intrinsically chiral metal cores. The results of our study will be expected to provide valuable tools for understanding the geometric and electronic origins of the chiroptical activities of metal clusters. Recently, not just icosahedral Au_13_ cores but also metal counterparts of the Au_13_ core (*i.e.*, M_13_ cores) have been highlighted as building blocks of ligand-protected metal clusters.^20^ Since these icosahedral M_13_ cores, with two stacking M_5_ rings, possess ideal topologies for generating strong charge rotations, the exploration of novel chiroptical metal clusters based on ubiquitous M_13_-based cores is worthy of further investigation.

## Conflicts of interest

There are no conflicts to declare.

## Supplementary Material

NA-003-D0NA00833H-s001

NA-003-D0NA00833H-s002
