# Supplementary material for: Chiroptical activity of Au13 clusters: experimental and theoretical understanding of the origin of helical charge movements
Source: Nanoscale Adv. 2020 Nov 5;3(4):1005–11. doi: 10.1039/d0na00833h (PMC9416943; doi:10.1039/d0na00833h)
Supplement: NA-003-D0NA00833H-s001 [file NA-003-D0NA00833H-s001.pdf]

Electronic Supplementary Material (ESI) for Nanoscale Advances.
This journal is © The Royal Society of Chemistry 2020

SUPPORTING INFORMATION

Chiroptical activity of Au_{13} clusters: experimental and theoretical understandings of the origin of helical charge movements

Yukatsu Shichibu,*,†,‡ Yuri Ogawa,† Mizuho Sugiuchi,† and Katsuaki Konishi*,†,‡

†Graduate School of Environmental Science and ‡Faculty of Environmental Earth Science, Hokkaido University, North 10 West 5, Sapporo 060-0810 Japan.

I. Experimental Details

A. Materials

Methanol (99.5%), acetonitrile (99%), and diethyl ether (99%) were purchased from Kanto Chemical Co., Inc. Ethanol (99%), tetramethylammonium chloride, and potassium hexafluorophosphate were obtained from Fujifilm Wako Pure Chemical Corporation. Sodium borohydride was obtained from TCI Co., Ltd. (*R*,*R*)-1,2-Bis[(2-methoxyphenyl)(phenylphosphino)]ethane (*R*,*R* -DIPAMP, ≥95%) and (*S*,*S*)-1,2-bis[(2-methoxyphenyl)(phenylphosphino)]ethane (*S*,*S* -DIPAMP, 95%) were obtained from Sigma-Aldrich. All reagents were used as received. The precursors Au_2(*R*,*R*-DIPAMP)Cl_2 and Au_2(*S*,*S*-DIPAMP)Cl_2 were prepared from AuCl(SMe_2) according to the literature with some modifications.[1,2]

B. Syntheses

[Au_{13}(*R*,*R*-DIPAMP)$_5Cl_2$]$(PF_6)_3$ (**2-*R***·$(PF_6)_3$): Tetramethylammonium chloride (77.8 mg, 710 μmol) and Au_2(*R*,*R*-DIPAMP)Cl_2 (65.6 mg, 71 μmol) were added to ethanol (12 mL), and the mixture, to which an ethanolic solution (1 mL) of sodium borohydride (5.4 mg, 140 μmol) was added dropwise, was stirred for 3 h at 25 °C. Subsequently, the resulting brownish solution was treated with 12 N HCl (125 μL), and stirring continued for an additional 24 h. After this time, the resulting mixture was dried in vacuo, the residue was redissolved in methanol, treated with excess potassium hexafluorophosphate to give a red precipitate, which was collected by filtration. The red solid was then washed with diethyl ether and methanol, and further purified by vapor diffusion of diethyl ether into an acetonitrile solution to give **2-*R***·$(PF_6)_3$ as red crystals (17.6 mg, 30 % based on Au). ESI-MS (m/z): 1641.2 ($[M–3PF_6]^{3+}$). Elemental analysis: calcd (%) for **2-*R***·$(PF_6)_3$ ($C_{140}H_{140}Au_{13}Cl_2F_{18}O_{10}P_{13}$): C 31.38, H 2.63; found: C 31.00, H 2.53; no nitrogen was found. The antipode (**2-*S***·$(PF_6)_3$) was prepared (21.1 mg, 36 % based on Au) and characterized similarly. Elemental analysis: calcd (%) for **2-*S***·$(PF_6)_3$ ($C_{140}H_{140}Au_{13}Cl_2F_{18}O_{10}P_{13}$): C 31.38, H 2.63; found: C 31.12, H 2.46; no nitrogen was found.

C. Measurements

Optical absorption and circular dichroism spectra were recorded using JASCO V-670 and JASCO J-760 spectrometers, respectively. Circularly polarized luminescence and photoluminescence spectra (uncorrected) were measured using JASCO CPL-300 spectrometer. Corrected photoluminescence spectra were obtained using JASCO FP-8700 spectrometer. Photoluminescence quantum yields were determined relative to anthracene in ethanol. Crystal structure data were collected on a Bruker SMART Apex II CCD diffractometer with graphite-monochromated Mo Kα radiation (λ = 0.71073 Å). All crystal structures were solved by direct methods and refined by full-matrix least-squares methods on F^2 using APEX 2 software. Non-hydrogen atoms were refined anisotropically. Hydrogen atoms were located at calculated positions and refined isotropically. ESI mass spectrometry was performed using a Bruker micrOTOF-HS mass spectrometer.

II. Computational Details

All calculations were performed using density functional theory (DFT) and time-dependent DFT (TDDFT) methods with a TURBOMOLE package.[3] The cluster model $[Au_{13}(PH_3)_{10}Cl_2]^{3+}$ (θ = 36°) was obtained from a ground-state geometry-optimization calculation with the crystallographically determined structure $[Au_{13}(PMe_2Ph)_{10}Cl_2]^{3+}$,[4] following a replacement of the hydrocarbon groups with hydrogen atoms. The other structure models of $[Au_{13}(PH_3)_{10}Cl_2]^{3+}$ with various torsion angles were built from the twisting of the two equatorial Au_5 rings with PH_3 ligands of the model (θ = 36°) around the axial axis. Ground- and excited-state calculations were performed at the B3LYP level of theory[5,6] using the def-SV(P) basis set.[7] The default 60-electron relativistic effective core potential (ECP)[8] was employed for the Au atoms, and the resolution of identity (RI) approximation for coulomb interactions[9] was used to speed up the calculations.

III. Experimental Results

A. ORTEP Drawing of 2-*R*·$(PF_6)_3$

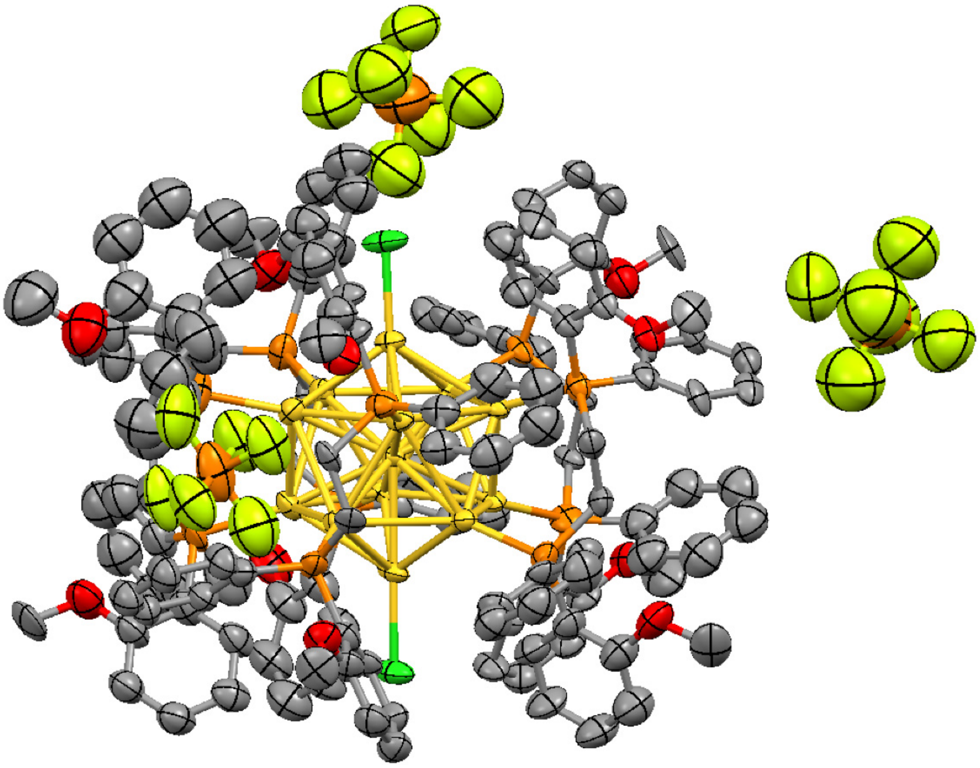

Fig. S1. ORTEP drawing for **2-***R*·$(PF_6)_3$. Thermal ellipsoids are drawn at the 50% probability level. For clarity, hydrogen atoms are omitted.

B. Crystal Data of 2-*R*·$(PF_6)_3$

Table S1. Crystal data and structure refinement for **2-*R***·$(PF_6)_3$.

Empirical formula	$C_{140}H_{140}Au_{13}Cl_2F_{18}O_{10}P_{13}$	
Formula weight	5358.59 g/mol	
Temperature	90(0) K	
Wavelength	0.71073 Å	
Crystal size	0.40 x 0.45 x 0.63 mm^3	
Crystal system	Orthorhombic	
Space group	$P2_12_12_1$	
Unit cell dimensions	a = 18.902(2) Å	α = 90°
	b = 30.413(3) Å	β = 90°
	c = 31.212(3) Å	γ = 90°
Volume	17943.(3) $Å^3$	
Z	4	
Density (calculated)	1.984 Mg/m^3	
Absorption coefficient	10.792 mm^{-1}	
$F(000)$	9912	
Theta range for data collection	0.94 to 27.57°	
Index ranges	$-24 \leq h \leq 22$, $-28 \leq k \leq 39$, $-39 \leq l \leq 40$	
Reflections collected	104645	
Independent reflections	41260 [R(int) = 0.0753]	
Completeness to theta = 27.57°	99.7 %	
Absorption correction	Multi-scan	
Max. and min. transmission	0.0990 and 0.0560	
Refinement method	Full-matrix least-squares on F^2	
Data / restraints / parameters	41260 / 2818 / 1535	
Goodness-of-fit on F^2	1.020	
Reflections with $I > 2\sigma(I)$	30087	
Final R indices [$I > 2\sigma(I)$]	R_1 = 0.0747, wR_2 = 0.1617	
R indices (all data)	R_1 = 0.1109, wR_2 = 0.1785	
Largest diff. peak and hole	3.014 and -3.282 $eÅ^{-3}$	

C. ORTEP Drawing of 2-*S*·$(PF_6)_3$

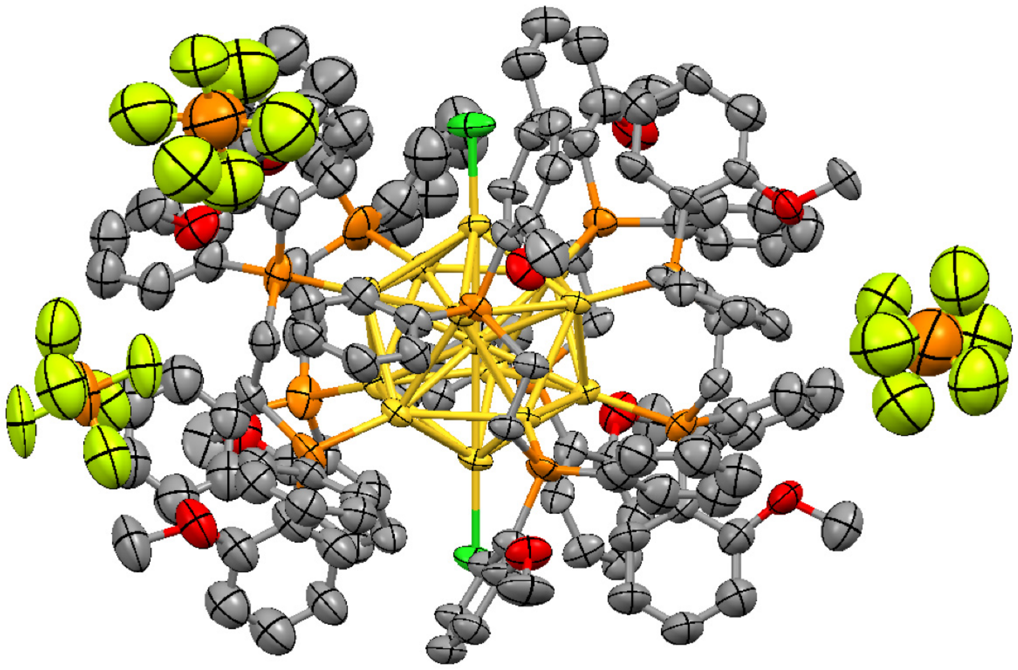

Fig. S2. ORTEP drawing for **2-*S***·$(PF_6)_3$. Thermal ellipsoids are drawn at the 50% probability level. For clarity, hydrogen atoms are omitted.

D. Crystal Data of 2-*S*·$(PF_6)_3$

Table S2. Crystal data and structure refinement for **2-*S***·$(PF_6)_3$.

Empirical formula	$C_{140}H_{140}Au_{13}Cl_2F_{18}O_{10}P_{13}$	
Formula weight	5358.59 g/mol	
Temperature	90(0) K	
Wavelength	0.71073 Å	
Crystal size	0.29 x 0.52 x 0.58 mm^3	
Crystal system	Orthorhombic	
Space group	$P2_12_12_1$	
Unit cell dimensions	a = 18.927(2) Å	$\alpha = 90°$
	b = 30.464(3) Å	$\beta = 90°$
	c = 31.269(4) Å	$\gamma = 90°$
Volume	18029.(3) $Å^3$	
Z	4	
Density (calculated)	1.974 Mg/m^3	
Absorption coefficient	10.741 mm^{-1}	
F(000)	9912	
Theta range for data collection	0.93 to 27.52°	
Index ranges	$-24 \leq h \leq 23$, $-39 \leq k \leq 37$, $-40 \leq l \leq 15$	
Reflections collected	104813	
Independent reflections	41247 [*R*(int) = 0.0811]	
Completeness to theta = 27.52°	99.7 %	
Absorption correction	Multi-scan	
Max. and min. transmission	0.1470 and 0.0620	
Refinement method	Full-matrix least-squares on F^2	
Data / restraints / parameters	41247 / 2626 / 1536	
Goodness-of-fit on F^2	1.023	
Reflections with $I > 2\sigma(I)$	28138	
Final *R* indices [$I > 2\sigma(I)$]	$R_1 = 0.0799$, $wR_2 = 0.1670$	
R indices (all data)	$R_1 = 0.1288$, $wR_2 = 0.1899$	
Largest diff. peak and hole	4.033 and -2.925 $eÅ^{-3}$	

E. Angles and Distances

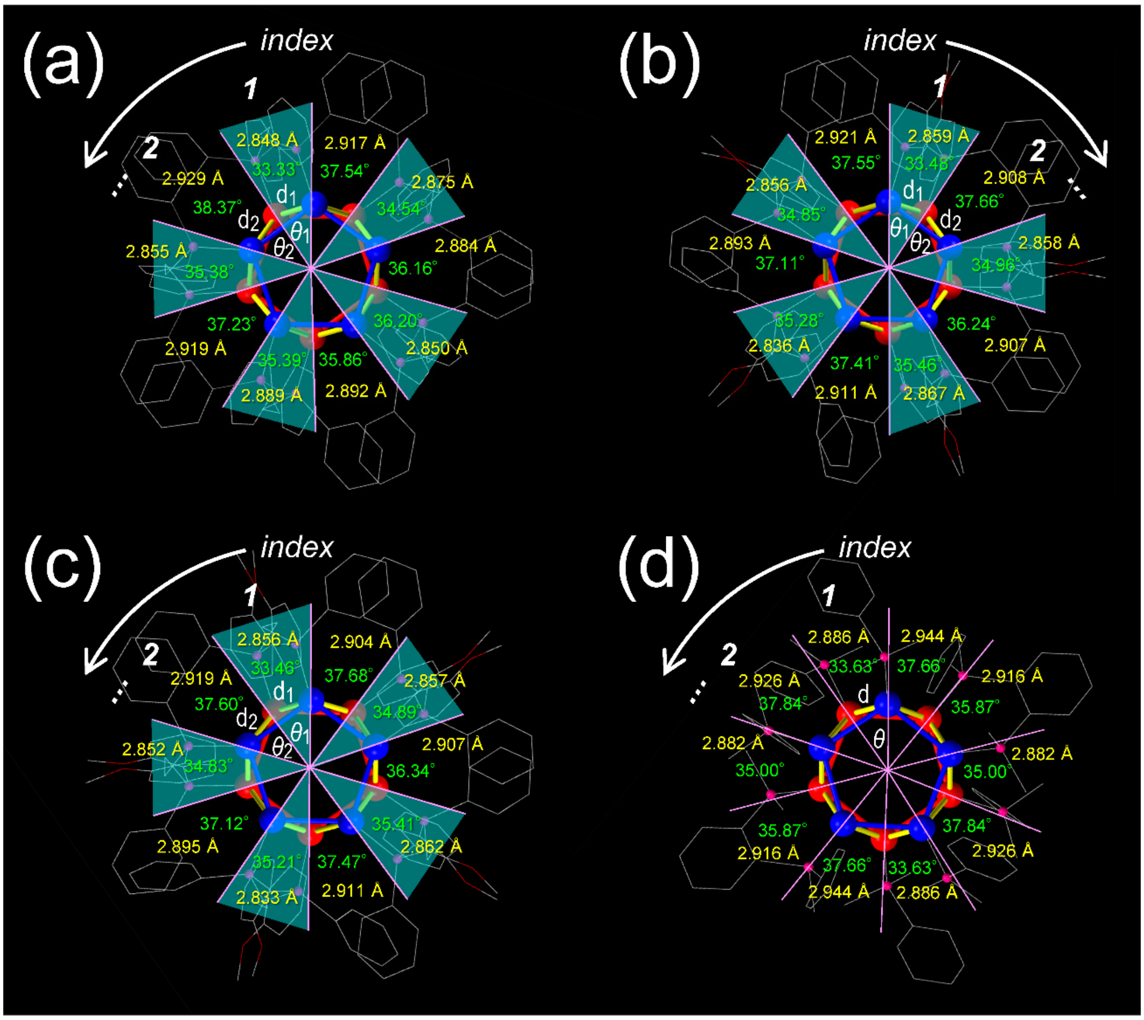

Fig. S3. Angles (θ_1 and θ_2) and distances (d_1 and d_2) of pentagonal anti-prism units of (a) **1**, (b) **2**-*R*, (c) **2**-*S*, and (d) $[Au_{13}(PMe_2Ph)_{10}Cl_2]^{3+}$. Areas relating to cross-linked diphosphines are shaded in aqua.

F. Photoluminescence Spectra

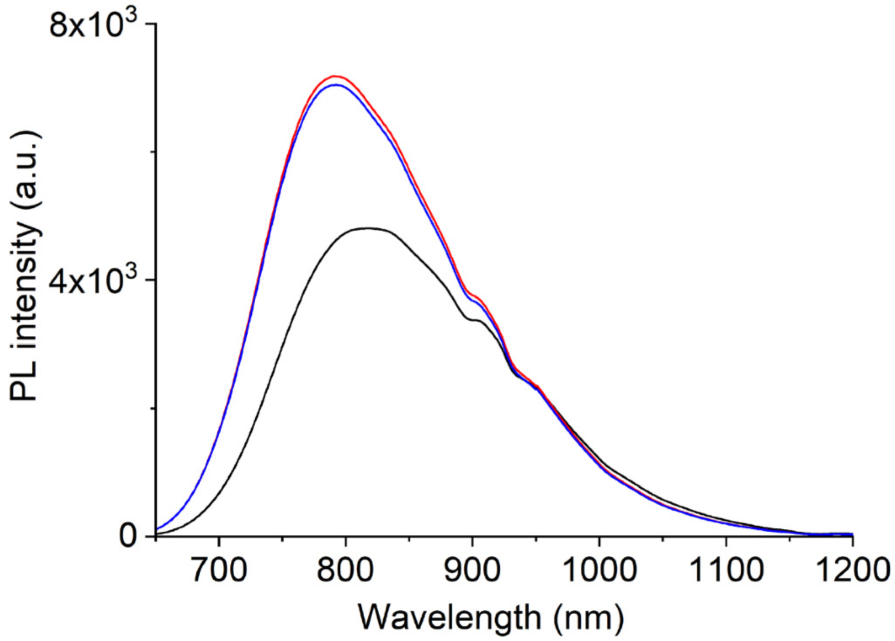

Fig. S4. Photoluminescence spectra (λ_{ex} = 495 nm) of the PF_6 salts of **1** (black), **2-*R*** (red) and **2-*S*** (blue) in acetonitrile.

IV. Computational Results

A. Absorption Spectra

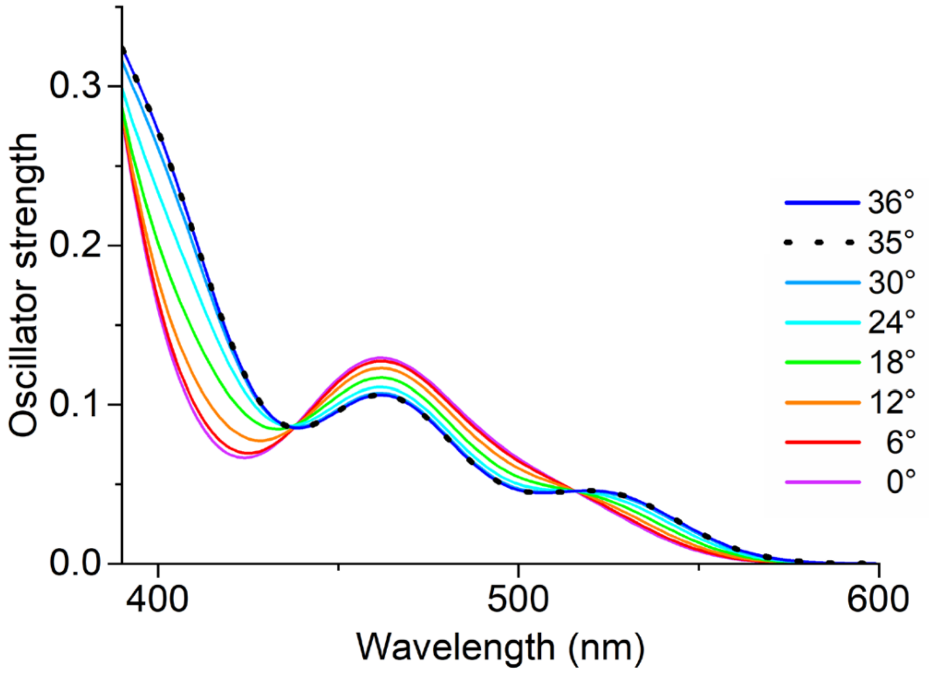

Fig. S5. Calculated absorption of $[Au_{13}(PH_3)_{10}Cl_2]^{3+}$ on the variation of θ from 36° to 0°.

B. Molecular Orbital and Transition Data

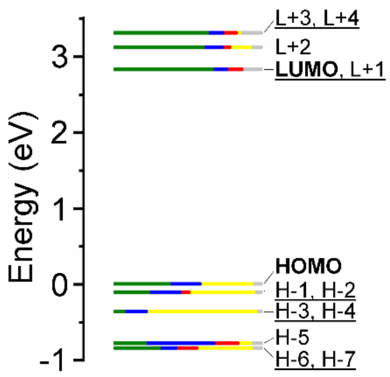

Fig. S6. Kohn-Sham (KS) orbital energy level diagram of $[Au_{13}(PH_3)_{10}Cl_2]^{3+}$ (θ = 18°). KS orbital is relative to the HOMO energy and is drawn to indicate the relative contributions (line length with color labels) of the atomic orbitals of Au (6sp) in green, Au (5d) in blue, P (3p) in red, Cl (3p) in yellow and miscellaneous in gray. H – X and L + Y represent HOMO – X and LUMO + Y, respectively.

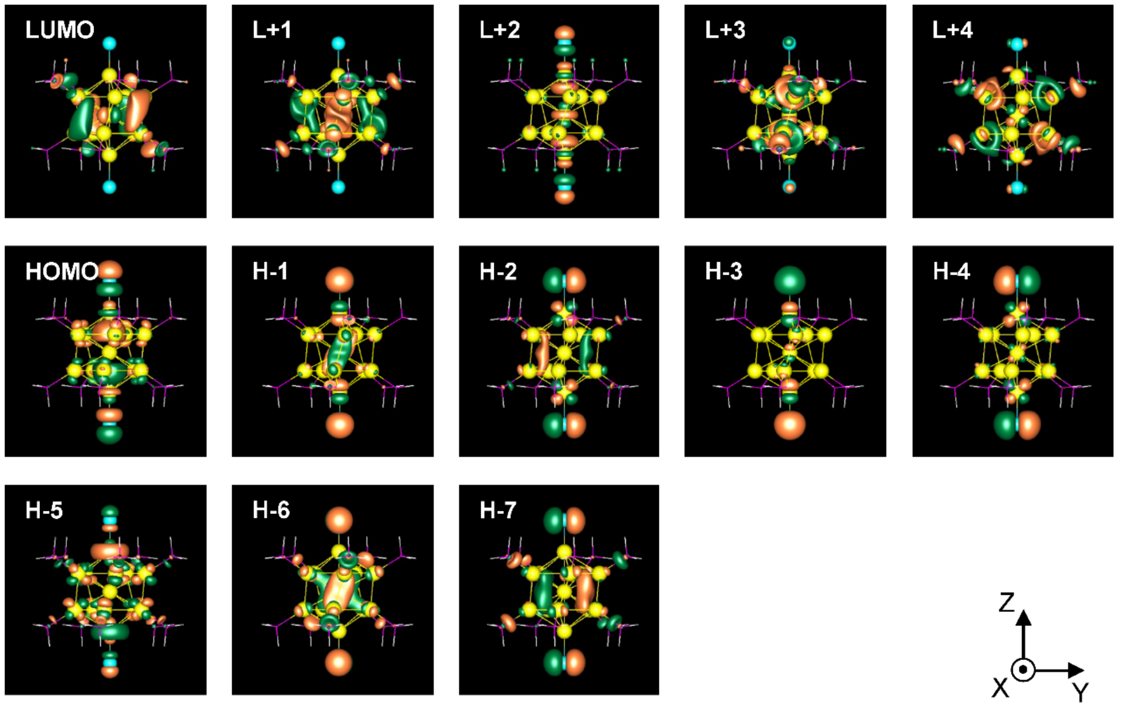

Fig. S7. Selected molecular orbitals of $[Au_{13}(PH_3)_{10}Cl_2]^{3+}$ (θ = 18°).

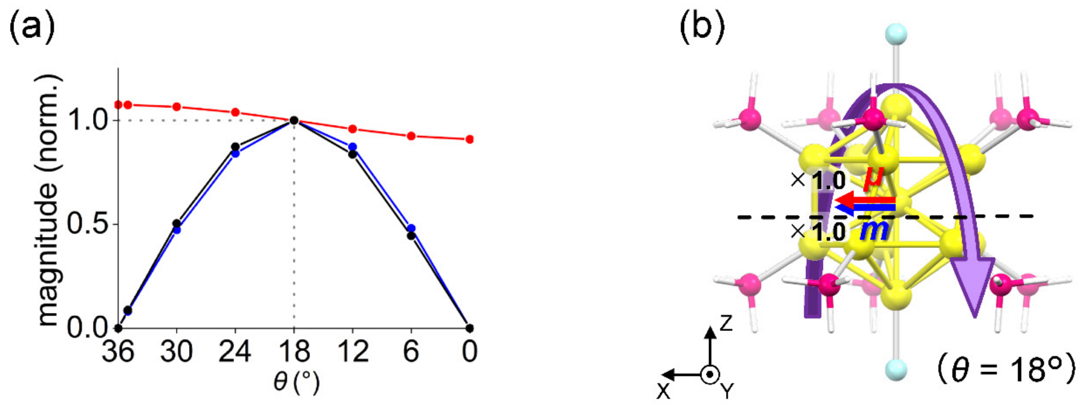

Fig. S8. (a) Normalized magnitudes of the rotatory strength (black), electric transition dipole moment (red) and magnetic transition dipole moment (blue) at $36° \geq \theta \geq 0°$, and (b) the electric (red) and magnetic (blue) transition dipole moments at $\theta = 18°$ for excitation I_x of $[Au_{13}(PH_3)_{10}Cl_2]^{3+}$. In part b, schematic representation of the helical charge movement is shown as a helical purple arrow, the magnitudes of the moments relative to those for excitation I_y are given near the respective arrow tips, and dashed line indicates the position of the *xy*-cutplanes given in Fig. S11.

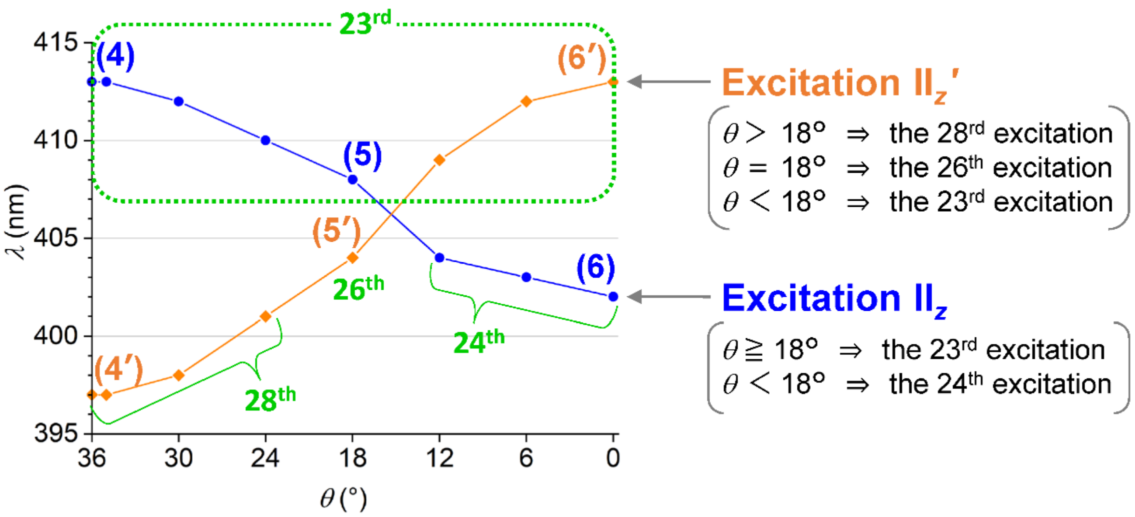

Fig. S9. Transition wavelengths of two types of the *z*-axis-oriented excitations (excitations II_z and II_z') upon variation in θ from 36° to 0° (blue and orange lines). The orders of singlet excitations are given in light green. The entry numbers in Tables 2 and S9 are given in parentheses.

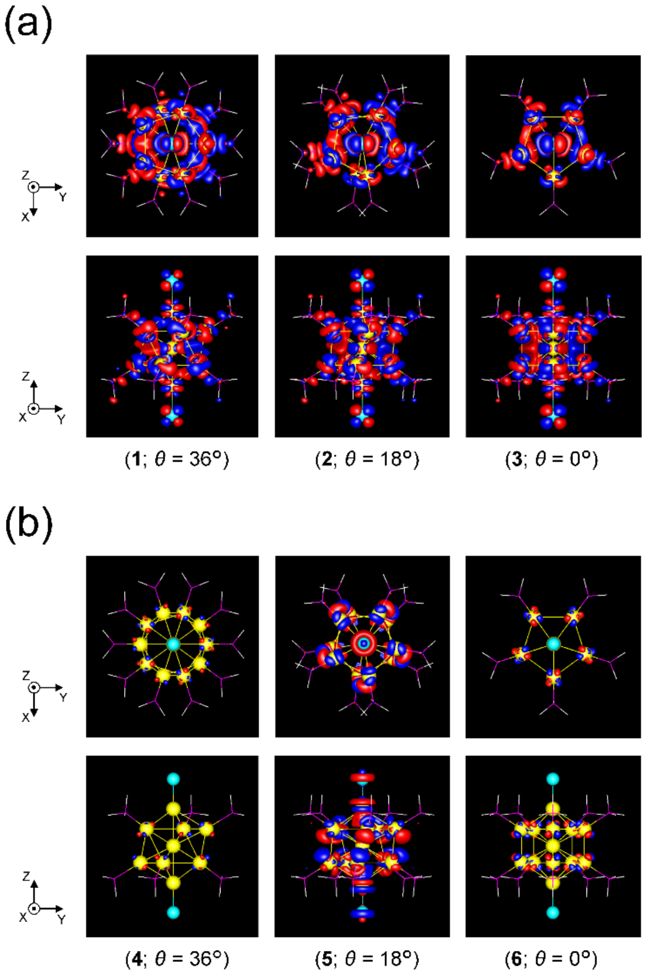

Fig. S10. Overall views of the transition densities for (a) excitation I_y and (b) excitation II_z of $[Au_{13}(PH_3)_{10}Cl_2]^{3+}$ at three representative θ. Electron (red) and hole (blue) distributions are displayed with isovalues of ±0.0003 a.u.

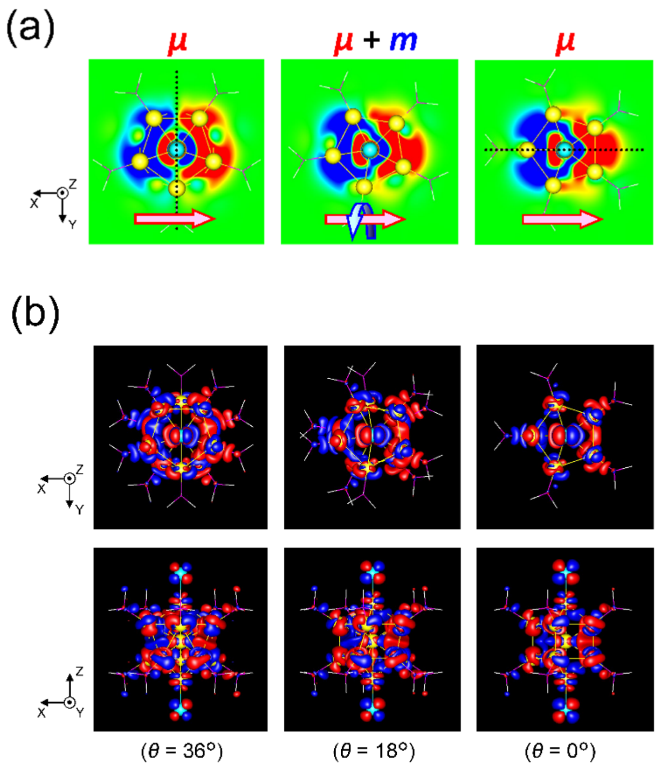

Fig. S11. (a) *xy*-Cutplane (within ±0.00015 a.u. range) and (b) overall (with ±0.0003 a.u. isovalues) views of the transition densities for excitation I_x of $[Au_{13}(PH_3)_{10}Cl_2]^{3+}$ at three representative θ. Red-colored and blue-colored areas represent electron increase and decrease during the electron excitation, respectively. The position of the *xy*-cutplanes in part a ($z = -0.25$) is provided as the dashed line in Fig. S8. Pink and aqua arrows schematically indicate charge translations and rotations, respectively.

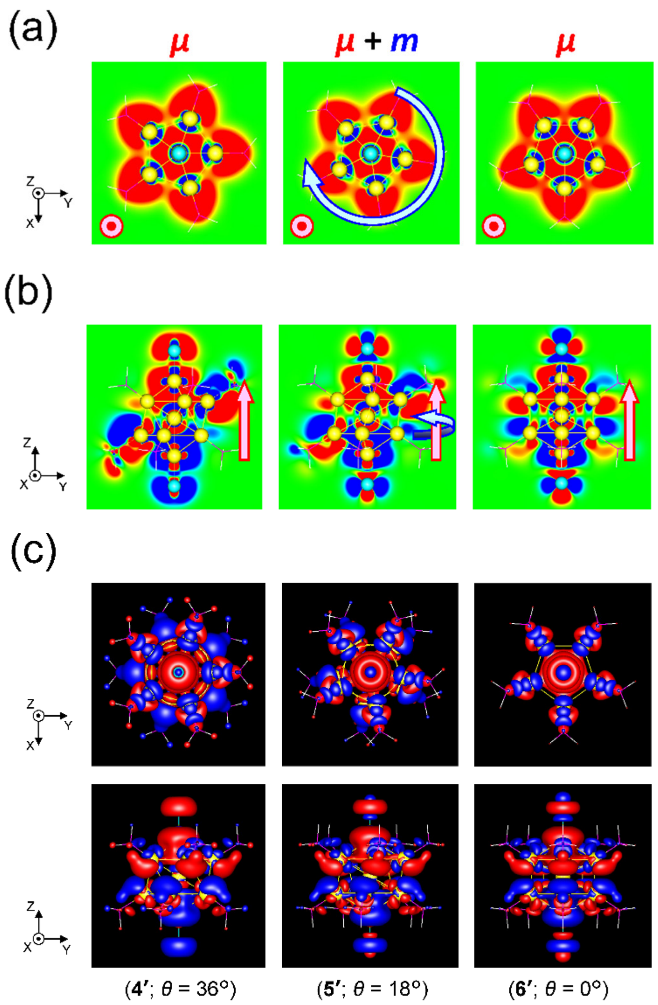

Fig. S12. (a) *xy*-Cutplane (z = +1.0; within ±0.00015 a.u. range), (b) *yz*-cutplane (x = 0.0; within ±0.00015 a.u. range) and (c) overall (with ±0.0003 a.u. isovalues) views of the transition densities for excitation II_z' of $[Au_{13}(PH_3)_{10}Cl_2]^{3+}$ at three representative θ. Red-colored and blue-colored areas represent electron increase and decrease during the electron excitation, respectively. The position of the *xy*-cutplanes in part a (z = +1.0) is provided as the dashed line in Fig. 5c(ii). Pink and aqua arrows schematically indicate charge translations and rotations, respectively.

Table S3. Torsion angles (θ), relative energies (ΔE), LUMO (E_L) and HOMO (E_H) energies, and HOMO–LUMO gap energies ($E_{H\text{-}L}$) of $[Au_{13}(PH_3)_{10}Cl_2]^{3+}$.

θ (°)	ΔE (eV)	E_L (eV) [a]	E_H (eV) [a]	$E_{H\text{-}L}$ (eV) [a]
36	0.00	-9.56	-12.41	2.85
35	0.00	-9.56 [0.00]	-12.41 [0.00]	2.85 [0.00]
30	0.06	-9.56 [0.00]	-12.40 [+0.01]	2.84 [−0.01]
24	0.26	-9.56 [0.00]	-12.39 [+0.02]	2.83 [−0.02]
18	0.57	-9.55 [+0.01]	-12.37 [+0.04]	2.82 [−0.03]
12	0.91	-9.55 [+0.01]	-12.36 [+0.05]	2.81 [−0.04]
6	1.20	-9.55 [+0.01]	-12.34 [+0.07]	2.79 [−0.06]
0	1.32	-9.55 [+0.01]	-12.34 [+0.07]	2.79 [−0.06]

[a] Differences from the value for $\theta = 36°$ are given in square brackets.

Table S4. Kohn-Sham orbitals, energies, and atomic orbital contributions of $[Au_{13}(PH_3)_{10}Cl_2]^{3+}$ ($\theta = 18°$).

MO	Orbital index	Orbital energy (eV) / Relative energy (eV)	% Au (*sp*)	% Au (*d*)	% P (*p*)	% Cl (*p*)
LUMO+4	234a	-9.07 / 3.30	64	10	10	2
LUMO+3	233a	-9.07 / 3.30	64	10	10	2
LUMO+2	232a	-9.25 / 3.11	61	13	5	14
LUMO+1	231a	-9.55 / 2.82	67	10	10	0
LUMO	230a	-9.55 / 2.82	67	10	10	0
HOMO	229a	-12.37 / 0.00	38	21	0	35
HOMO-1	228a	-12.48 / -0.11	24	21	6	44
HOMO-2	227a	-12.48 / -0.11	24	21	6	44
HOMO-3	226a	-12.74 / -0.36	7	15	0	74
HOMO-4	225a	-12.74 / -0.36	7	15	0	74
HOMO-5	224a	-13.15 / -0.78	22	47	16	8
HOMO-6	223a	-13.22 / -0.85	31	12	14	37
HOMO-7	222a	-13.22 / -0.85	31	12	14	37

Table S5. Excited states, energies, oscillator strengths, rotatory strengths and primary orbital-orbital transitions of $[Au_{13}(PH_3)_{10}Cl_2]^{3+}$ ($\theta = 18°$). H – X and L + Y represent HOMO – X and LUMO + Y, respectively.

Excitation index	Energy (eV) / Wavelength (nm)	Oscillator strength	Rotatory strength (10^{-40} esu$^2 \cdot$ cm^2)	Dominant transition	Nature of transition
1	2.16 / 575	2.149×10^{-10}	-1.439×10^{-6}	229a→230a	H→L
2	2.16 / 575	8.603×10^{-9}	6.598×10^{-6}	229a→231a	H→L+1
3	2.34 / 529	5.272×10^{-7}	1.574×10^{-4}	228a→230a, 227a→231a	H-1→L, H-2→L+1
4	2.34 / 529	1.439×10^{-8}	6.909×10^{-6}	227a→230a, 228a→231a	H-2→L, H-1→L+1
5	2.39 / 520	1.884×10^{-2}	5.126×10^{0}	228a→231a, 227a→230a	H-1→L+1, H-2→L
6	2.39 / 519	1.888×10^{-2}	5.095×10^{0}	227a→231a, 228a→230a	H-2→L+1, H-1→L
7	2.56 / 485	6.679×10^{-3}	1.722×10^{0}	228a→232a, 229a→233a	H-1→L+2, H→L+3
8	2.56 / 485	6.671×10^{-3}	1.715×10^{0}	227a→232a, 229a→234a	H-2→L+2, H→L+4
9	2.60 / 476	4.349×10^{-3}	-4.314×10^{-1}	229a→232a, 228a→233a, 227a→234a	H→L+2, H-1→L+3, H-2→L+4
10	2.64 / 470	1.920×10^{-7}	1.792×10^{-3}	226a→230a, 225a→231a	H-3→L, H-4→L+1
11	2.64 / 470	7.555×10^{-8}	6.966×10^{-4}	225a→230a, 226a→231a	H-4→L, H-3→L+1
12	2.64 / 469	2.281×10^{-4}	1.808×10^{0}	225a→231a, 226a→230a	H-4→L+1, H-3→L
13	2.64 / 469	2.257×10^{-4}	1.803×10^{0}	226a→231a, 225a→230a	H-3→L+1, H-4→L
14	2.69 / 460	5.137×10^{-2}	-1.663×10^{0}	229a→233a, 228a→232a	H→L+3, H-1→L+2
15	2.69 / 460	5.135×10^{-2}	-1.707×10^{0}	229a→234a, 227a→232a	H→L+4, H-2→L+2
16	2.75 / 451	1.735×10^{-8}	2.655×10^{-5}	228a→234a, 227a→233a	H-1→L+4, H-2→L+3
17	2.77 / 448	6.790×10^{-8}	-6.370×10^{-5}	227a→233a, 228a→234a	H-2→L+3, H-1→L+4
18	2.77 / 448	4.516×10^{-7}	-2.555×10^{-4}	227a→234a, 228a→233a	H-2→L+4, H-1→L+3
19	2.81 / 441	3.373×10^{-4}	-6.450×10^{0}	226a→232a	H-3→L+2
20	2.81 / 441	3.378×10^{-4}	-6.454×10^{0}	225a→232a	H-4→L+2
21	2.95 / 420	1.562×10^{-8}	8.238×10^{-6}	224a→230a	H-5→L
22	2.95 / 420	1.428×10^{-8}	6.761×10^{-5}	224a→231a	H-5→L+1
23	3.04 / 408	1.348×10^{-2}	-4.787×10^{1}	225a→233a, 226a→234a	H-4→L+3, H-3→L+4
24	3.06 / 406	6.714×10^{-7}	-1.979×10^{-4}	225a→233a, 226a→234a, 223a→230a, 222a→231a	H-4→L+3, H-3→L+4, H-6→L, H-7→L+1
25	3.06 / 406	1.064×10^{-9}	-9.290×10^{-7}	226a→233a, 225a→234a, 223a→231a, 222a→230a	H-3→L+3, H-4→L+4, H-6→L+1, H-7→L
26	3.07 / 404	4.539×10^{-2}	-5.648×10^{0}	224a→232a, 227a→234a, 228a→233a	H-5→L+2, H-2→L+4, H-1→L+3
27	3.09 / 402	1.063×10^{-8}	1.806×10^{-5}	222a→230a, 223a→231a, 226a→233a, 225a→234a	H-7→L, H-6→L+1, H-3→L+3, H-4→L+4
28	3.09 / 402	3.671×10^{-7}	-5.642×10^{-5}	223a→230a, 222a→231a, 226a→234a, 225a→233a	H-6→L, H-7→L+1, H-3→L+4, H-4→L+3
29	3.16 / 393	4.180×10^{-2}	2.852×10^{0}	223a→231a, 222a→230a, 224a→233a	H-6→L+1, H-7→L, H-5→L+3
30	3.16 / 393	4.192×10^{-2}	2.787×10^{0}	222a→231a, 223a→230a, 224a→234a	H-7→L+1, H-6→L, H-5→L+4

Table S6. Torsion angles (θ), transition wavelengths (λ), rotatory strengths (R), magnitudes of electric transition dipole moments ($|\boldsymbol{\mu}|$) and magnitudes of magnetic transition dipole moments ($|\boldsymbol{m}|$) for excitation I_x of $[Au_{13}(PH_3)_{10}Cl_2]^{3+}$.

θ (°)	λ (nm)	R (10^{-40} esu$^2\cdot$cm^2) ^{a}	$\|\boldsymbol{\mu}\|$ (D) ^{a}	$\|\boldsymbol{m}\|$ (μ_B) ^{a}
36	524	0.000 [0.000]	1.551 [1.075]	0.000 [0.000]
35	524	0.449 [0.088]	1.551 [1.075]	0.003 [0.081]
30	523	2.579 [0.503]	1.537 [1.065]	0.018 [0.472]
24	522	4.475 [0.873]	1.498 [1.038]	0.032 [0.841]
18	520	5.126 [1.000]	1.443 [1.000]	0.038 [1.000]
12	517	4.287 [0.836]	1.383 [0.959]	0.033 [0.872]
6	516	2.274 [0.444]	1.334 [0.924]	0.018 [0.480]
0	515	0.000 [0.000]	1.311 [0.909]	0.000 [0.000]

^{a} Normalized magnitudes against the magnitude for θ = 18° are given in square brackets.

Table S7. Torsion angles (θ), transition wavelengths (λ), rotatory strengths (R), magnitudes of electric transition dipole moments ($|\boldsymbol{\mu}|$) and magnitudes of magnetic transition dipole moments ($|\boldsymbol{m}|$) for excitation I_y of $[Au_{13}(PH_3)_{10}Cl_2]^{3+}$.

θ (°)	λ (nm)	R (10^{-40} esu$^2\cdot$cm^2) ^{a}	$\|\boldsymbol{\mu}\|$ (D) ^{a}	$\|\boldsymbol{m}\|$ (μ_B) ^{a}
36	524	0.000 [0.000]	1.552 [1.074]	0.000 [0.000]
35	524	0.444 [0.087]	1.551 [1.074]	0.003 [0.081]
30	523	2.555 [0.501]	1.538 [1.065]	0.018 [0.471]
24	522	4.443 [0.872]	1.499 [1.038]	0.032 [0.840]
18	519	5.095 [1.000]	1.444 [1.000]	0.038 [1.000]
12	517	4.262 [0.837]	1.386 [0.959]	0.033 [0.872]
6	516	2.261 [0.444]	1.337 [0.926]	0.018 [0.480]
0	515	0.000 [0.000]	1.313 [0.909]	0.000 [0.000]

^{a} Normalized magnitudes against the magnitude for θ = 18° are given in square brackets.

Table S8. Torsion angles (θ), transition wavelengths (λ), rotatory strengths (R), magnitudes of electric transition dipole moments ($|\boldsymbol{\mu}|$) and magnitudes of magnetic transition dipole moments ($|\boldsymbol{m}|$) for excitation II_z of $[Au_{13}(PH_3)_{10}Cl_2]^{3+}$.

θ (°)	λ (nm)	R (10^{-40} esu$^2\cdot$cm^2) ^{a}	$\|\boldsymbol{\mu}\|$ (D) ^{a}	$\|\boldsymbol{m}\|$ (μ_B) ^{a}
36	413	0.000 [0.000]	0.000 [0.000]	0.459 [0.962]
35	413	-1.524 [0.032]	0.036 [0.033]	0.459 [0.962]
30	412	-9.428 [0.197]	0.221 [0.204]	0.460 [0.964]
24	410	-21.27 [0.444]	0.493 [0.456]	0.465 [0.976]
18	408	-47.87 [1.000]	1.082 [1.000]	0.477 [1.000]
12	404	10.36 [0.217]	0.349 [0.322]	0.321 [0.672]
6	403	0.494 [0.010]	0.015 [0.014]	0.357 [0.748]
0	402	0.000 [0.000]	0.000 [0.000]	0.356 [0.747]

^{a} Normalized magnitudes against the magnitude for θ = 18° are given in square brackets.

Table S9. Selected singlet excitation data for $[Au_{13}(PH_3)_{10}Cl_2]^{3+}$.

Entry	θ (°)	λ (nm)	R (10^{-40} esu$^2 \cdot$ cm^2)	$\|\boldsymbol{\mu}\|$ (D)	$\|\boldsymbol{m}\|$ (μ_B)
**Excitation II$_z$'**					
4'	36	397	0.000	3.304	0.000
5'	18	404	-5.648	1.975	0.031
6'	0	413	0.000	1.145	0.001

References

[1] M. C. Brandys, M. C. Jennings and R. J. Puddephatt, *J. Chem. Soc.*, *Dalton Trans.*, 2000, 4601-4606.
[2] Y. Shichibu, K. Suzuki and K. Konishi, *Nanoscale*, 2012, **4**, 4125-4129.
[3] TURBOMOLE V7.4 2019, a development of University of Karlsruhe and Forschungszentrum Karlsruhe GmbH, 1989-2007, TURBOMOLE GmbH, since 2007; available from http://www.turbomole.com.
[4] C. E. Briant, B. R. C. Theobald, J. W. White, L. K. Bell and D. M. P. Mingos, *J. Chem. Soc., Chem. Commun.*, 1981, 201-202.
[5] C. Lee, W. Yang and R. G. Parr, *Phys. Rev. B*, 1988, **37**, 785-789.
[6] A. D. Becke, *J. Chem. Phys.*, 1993, **98**, 5648-5652.
[7] A. Schäfer, H. Horn and R. Ahlrichs, *J. Chem. Phys.*, 1992, **97**, 2571-2577.
[8] D. Andrae, U. Häußermann, M. Dolg, H. Stoll and H. Preuß, *Theor. Chim. Acta*, 1990, **77**, 123-141.
[9] R. Ahlrichs, M. Bär, M. Häser, H. Horn and C. Kölmel, *Chem. Phys. Lett.,* 1989, **162**, 165-169.
